# The *Lancet Global Health* Commission on Global Eye Health: key findings

**Published:** 2021-07-20

**Authors:** Hugh Bassett, Hannah Faal, Matthew Burton

**Affiliations:** 1 Communications Officer: London School of Hygiene & Tropical Medicine, International Centre for Eye Health, London.; 2 Professor of International Eye Health: University of Calabar, Calabar, Nigeria.; 3 Director: International Centre for Eye Health, London School of Hygiene and Tropical Medicine, London, UK.

**More than 70 leading figures from 25 countries have contributed to the *Lancet Global Health* Commission on Global Eye Health – a wide-ranging report synthesising new and existing research across many aspects of eye health which was published in April 2021. This article series will look at the findings of the Commission in more depth, starting with a focus on the key findings.**

Where to access the report: **https://globaleyehealth commission.org**

**Figure:**
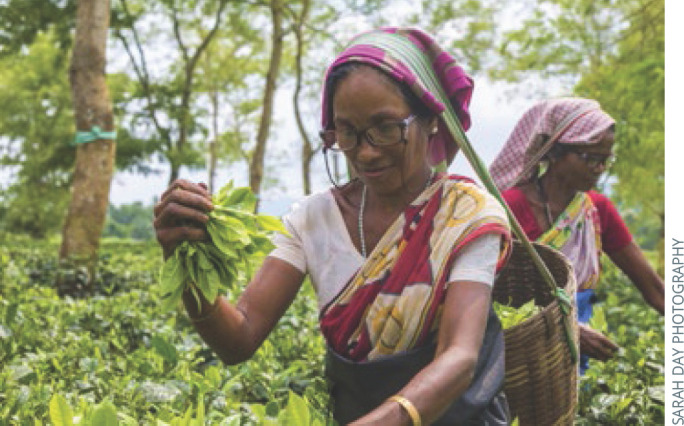
Investing in eye health interventions, such as spectacles for near vision, has been shown to boost productivity. **INDIA**

February this year saw the publication of the ***Lancet Global Health* Commission on Global Eye Health**. The Commission is the work of an interdisciplinary group of 73 academics and national programme leaders and practitioners from 25 countries, co-chaired by Professor Matthew Burton from the **International Centre for Eye Health** (publishers of the *Community Eye Health Journal*) and Professor Hannah Faal from the University of Calabar, Nigeria.

## Key findings

In 2020, 1.1 billion people were living with vision impairment, including blindness, and hundreds of millions more have ongoing eye care needs. Unless governments begin investing more in the people, equipment, and systems needed to deliver eye care, there could be 1.8 billion people living with vision impairment by 2050.

**Figure 1:**
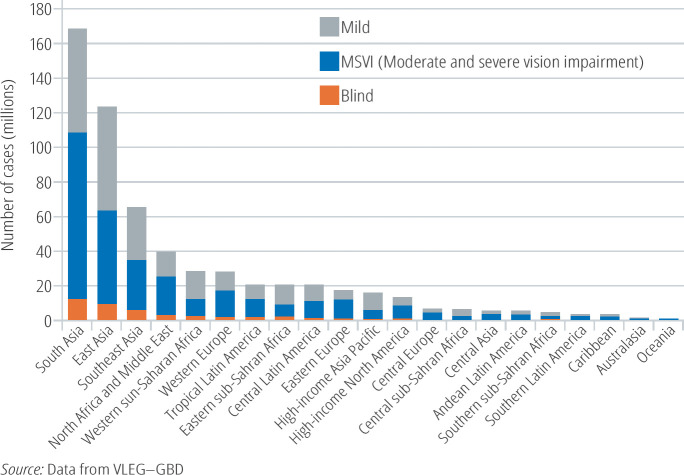
Vision impairment by Global Burden of Disease region: number of people with vision impairment.

Blindness and vision impairment does not affect everyone equally. An estimated 90% of people with vision impairment live in low- and middle-income countries (Figure 1). Women, people living in rural areas, and people belonging to ethnic minority groups are also more likely to be affected.

The scale of this unaddressed need leads to a large economic cost globally, with analyses indicating that blindness, together with moderate to severe vision loss, currently results in US $411 billion in lost economic productivity.

This is despite the fact that there are highly cost-effective treatments for most causes of vision impairment. In fact, over 90% of people living with vision loss would be able to see clearly after receiving cataract surgery or a pair of spectacles. Both interventions are highlighted in the report as being highly cost effective in many settings, particularly in resource-limited settings.

The Commission also examined the evidence that interventions to improve eye health can help to advance several of the United Nations’ Sustainable Development Goals. It highlights clear examples, such as the impact of cataract surgery on poverty reduction (SDG1) and the improvement of educational attainment (SDG4) following the provision of glasses to children with refractive error.

New thinking is needed to increase investment in eye health. In many countries there is a need to include eye health services in general health care, as part of **Universal Health Coverage**. This will require the integration of eye health into national health policies, financing, and health workforce planning.

Eye care services also need to be delivered as close to the population as possible. To do this, eye health must be included in general primary health services and secondary eye services must be strengthened. Working with communities as co-producers of health, expanding the eye health workforce to meet population needs, and integrating eye health teams into the general health care workforce can be powerful tools to strengthen eye care delivery and improve lives.

The rights of people living with vision impairment should be championed. We can do this by creating a more inclusive society and providing rehabilitation services, assistive technology, and accessible spaces. To help achieve immediate and substantial benefits for societies and people living with vision impairment, the report’s authors call on governments to urgently invest in eye health and to start rapidly improving lives and livelihoods by putting eye care in its rightful place on the global public health agenda.

